# Soil salinity modulates fatty acid composition and antioxidant capacity of rice bran oil

**DOI:** 10.7717/peerj.21476

**Published:** 2026-07-02

**Authors:** Jinnawat Manasathien, Woraporn Laojinda, Jakrada Attarataya, Piyanut Khanema

**Affiliations:** 1 Program of Biology, Faculty of Science and Technology, Nakhon Ratchasima Rajabhat University, Nakhon Ratchasima, Thailand; 2 Faculty of Science, Mahasarakham University, Maha Sarakham, Thailand; 3 Beamline Division, Synchrotron Light Research Institute (Public Organization), Nakhon Ratchasima, Thailand; 4 Department of Biology, Faculty of Science, Mahasarakham University, Maha Sarakham, Thailand; 5 Isan Saline Soil Research Unit (ISSRU), Faculty of Science, Mahasarakham University, Maha Sarakham, Thailand

**Keywords:** Antioxidant activity, Fatty acid composition, Rice bran oil, Soil salinity, γ-oryzanol, Unsaturated fatty acids, Multivariate analysis, Field-based study, Rice cultivars

## Abstract

**Background:**

Rice bran oil (RBO) is valued for its nutritional and functional properties; however, the influence of soil salinity on RBO quality under field conditions remains insufficiently understood. This study examined RBO derived from two major Thai rice varieties, glutinous RD6 and non-glutinous KDML105, cultivated across saline-affected sites along the Siao Yai River in northeastern Thailand.

**Methods:**

Soil chemical properties, including electrical conductivity of the saturated paste extract (EC_e_), sodium adsorption ratio (SAR), exchangeable cations, and chloride, were evaluated in relation to RBO yield, γ-oryzanol content, antioxidant activity, elemental composition, fatty acid profiles, and synchrotron radiation–based Fourier transform infrared microspectroscopy (SR-FTIR) characteristics.

**Results:**

Soil salinity was strongly associated with variations in RBO compositional quality. Significant salinity effects were observed for specific saturated fatty acids, particularly stearic (C18:0) and arachidic acids (C20:0), which decreased under high-salinity conditions, and linoleic acid (C18:2n6c), which increased under high salinity. In addition, total polyunsaturated fatty acids (ΣPUFA) and fatty acid ratios, including monounsaturated fatty acids/saturated fatty acids (MUFA/SFA), polyunsaturated fatty acids/saturated fatty acids (PUFA/SFA), and unsaturated fatty acids/saturated fatty acids (UFA/SFA) were significantly higher under saline conditions, indicating a shift toward a relatively more unsaturated lipid profile. Antioxidant activity increased significantly with salinity and was positively associated with salinity indicators and unsaturated fatty acids, while carotenoid content was also higher under saline conditions and γ-oryzanol showed a variety-dependent response to salinity. In contrast, RBO yield was not affected by salinity, suggesting a potential decoupling between oil quantity and compositional quality, although this relationship warrants further controlled investigation. Calcium concentration in RBO increased under high salinity, suggesting a soil–oil linkage, whereas other elements showed limited responses. SR-FTIR analysis revealed comparable triacylglycerol spectral features across all samples, indicating that salinity-related differences were quantitative rather than structural.

**Conclusion:**

These findings suggest that saline soil environments may enhance key nutritional and functional attributes of RBO without compromising oil yield, highlighting opportunities for valorizing RBO produced in saline-prone agricultural regions.

## Introduction

Rice bran oil (RBO) is increasingly recognized as a high-value functional edible oil due to its balanced fatty acid composition and its rich content of bioactive compounds, including γ-oryzanol, tocopherols, tocotrienols, phenolics, and flavonoids. These constituents contribute to antioxidant capacity, cholesterol-lowering effects, and anti-inflammatory activity, positioning RBO as a nutritionally relevant oil with potential health-promoting properties beyond its conventional role as a cooking medium (Punia et al., 2021; Sahini & Mutegoa, 2023). Clinical studies further demonstrate that RBO consumption reduces serum total and LDL cholesterol levels, effects largely attributed to unsaponifiable components such as phytosterols and γ-oryzanol rather than fatty acid composition alone (Most et al., 2005). Recent reviews further highlight RBO as a multifunctional edible oil with broad nutritional, therapeutic, and industrial relevance, driven by its balanced fatty acid profile and diverse bioactive constituents (Tufail et al., 2024).

The nutritional quality and functional properties of RBO are shaped by multiple interacting factors, including rice genotype, agronomic conditions, environmental stressors, and extraction processes. Rice variety strongly influences fatty acid distribution and the abundance of health-related bioactive compounds (Wisetkomolmat et al., 2022), while environmental conditions can modulate grain metabolism and lipid biosynthesis. Among these factors, salinity is of particular interest because it induces osmotic and oxidative stress that can remodel membrane lipid composition, alter lipid metabolism, and modulate antioxidant responses in plants (Guo et al., 2022; Pan et al., 2023). Fatty acid desaturation pathways, including those involving fatty acid desaturase 3 (FAD3) and related desaturases, have also been implicated in stress adaptation (Gishini, Kachroo & Hildebrand, 2025). In addition, extraction and pretreatment strategies can affect oil yield, oxidative stability, and phytochemical retention, highlighting the need to consider environmental effects alongside technological factors when evaluating RBO quality (Mathias et al., 2023; Pimpa, Thongraung & Sutthirak, 2021).

Although salinity is commonly viewed as a constraint on crop productivity, increasing evidence suggests that it may also act as a biochemical regulator influencing the nutritional and functional quality of plant-derived oils. Salinity-induced lipid desaturation, leading to higher proportions of unsaturated fatty acids, has been reported in rice grains and other cereals as an adaptive response to maintain membrane fluidity under stress (Razzaq et al., 2020). From a food science perspective, such shifts are desirable because monounsaturated fatty acids, particularly oleic acid, enhance oxidative stability, shelf life, and health attributes of edible oils (Most et al., 2005; Xu et al., 2021). However, field-based evidence directly linking soil salinity gradients to the functional quality of RBO remains limited, particularly under heterogeneous agronomic conditions.

Thailand provides a relevant context for addressing this knowledge gap, as RBO is widely promoted as a functional food ingredient and exhibits substantial variation in γ-oryzanol content, fatty acid composition, and antioxidant capacity among local rice varieties (Wisetkomolmat et al., 2022). Two economically important cultivars are RD6 (glutinous) and KDML105 (non-glutinous, jasmine-type), both widely cultivated in northeastern Thailand. The Siao Yai River basin presents a natural gradient of soil salinity and fertility across multiple districts, offering an opportunity to investigate how edaphic variation influences RBO quality under realistic field conditions.

Accordingly, this study investigated RBO extracted from RD6 and KDML105 cultivated across saline-affected sites along the Siao Yai River. We hypothesized that increasing soil salinity, reflected by elevated electrical conductivity of the saturated paste extract (EC_e_) and sodium adsorption ratio (SAR), would be associated with enhanced lipid desaturation toward unsaturated fatty acids and increased antioxidant accumulation in RBO, while preserving the fundamental triacylglycerol structure of the oil. The specific objectives were to (i) characterize fatty acid composition, antioxidant activity, and elemental profiles of RBO across contrasting salinity levels, (ii) compare responses between glutinous and non-glutinous rice varieties, and (iii) elucidate relationships between soil chemical properties and oil quality traits. By linking soil conditions to RBO nutritional and functional attributes, this study provides insight into how environmental factors shape food oil quality and supports the valorization of RBO produced in saline-prone agricultural systems.

## Materials & Methods

### Study area and sampling

**Figure 1:**
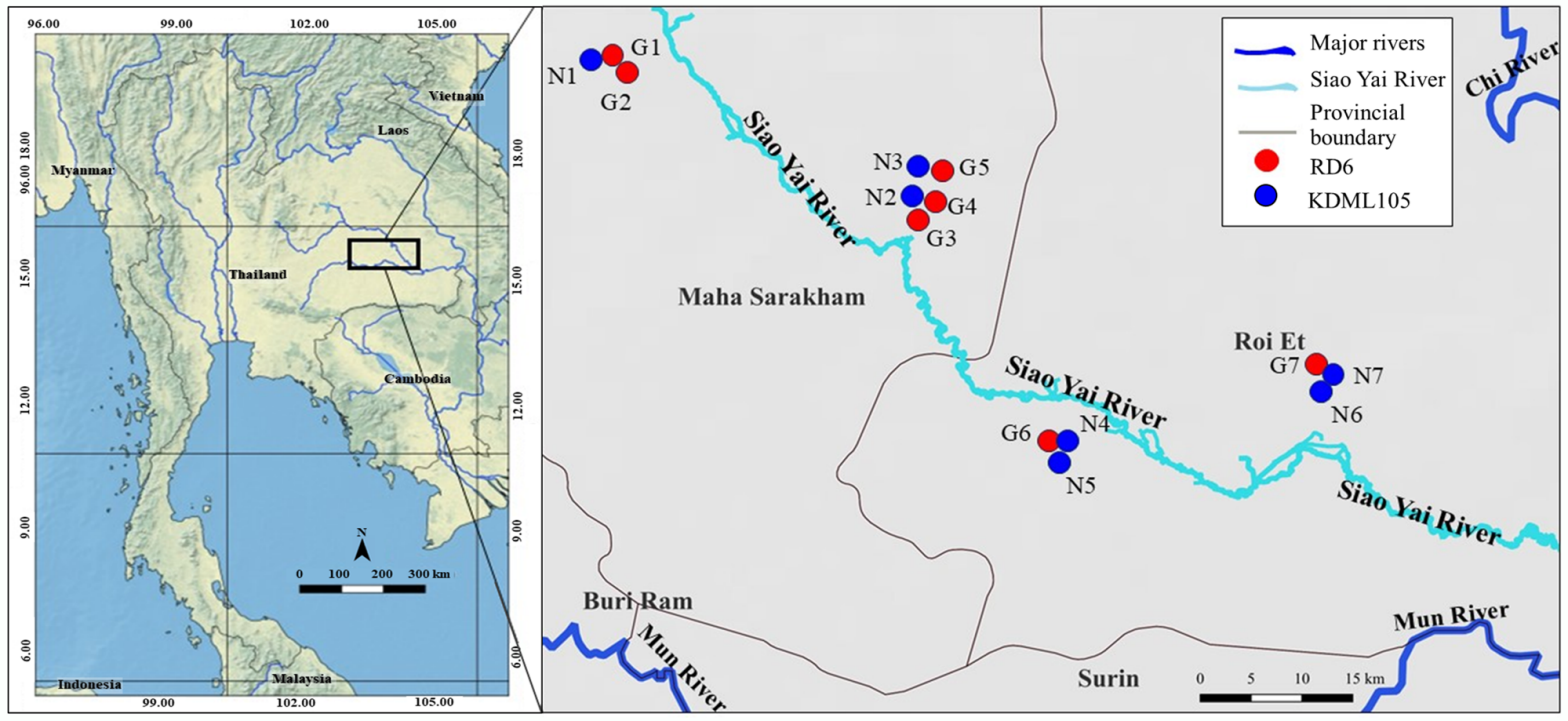
Study area and rice sampling-site locations in northeastern Thailand. The map shows the locations of 14 rice cultivation sites along the Siao Yai River basin in Maha Sarakham and Roi Et provinces, northeastern Thailand. Sampling sites include glutinous rice (RD6; red circles) and non-glutinous rice (KDML105; blue circles). Major rivers, the Siao Yai River, and provincial boundaries are shown. The map was generated by the authors in QGIS 3.44.9 using public domain Natural Earth datasets for the base map, river layers, and administrative boundaries. The Siao Yai River layer was manually created by the authors, and sampling-site coordinates were collected during field surveys.

Rice bran samples were collected in January 2025 from rice fields distributed along the Siao Yai River in northeastern Thailand, covering four districts—Borabue, Wapi Pathum, Kaset Wisai, and Suwannaphum—across Maha Sarakham and Roi Et provinces (Fig. 1). Two rice cultivars were selected: RD6 (glutinous) and KDML105 (non-glutinous, jasmine-type). At harvest, paddy rice from each site was milled, and freshly obtained bran was immediately collected, sealed in airtight bags, and stored at −20 °C until analysis. Soil samples were collected from the topsoil (0–25 cm) during the late vegetative to early reproductive stage of rice growth, when plants were actively exposed to site-specific salinity conditions. Rice cultivation at the sampled sites generally followed local farmer-managed practices typical of northeastern Thailand. Fertilizer management was not strictly standardized among fields, but farmers commonly used compound fertilizers under split-application schedules typical of Thai rice cultivation. Detailed field history and exact fertilizer inputs were not uniformly available for all sites; therefore, the results should be interpreted as field-based associations under naturally varying agronomic conditions.

### Soil chemical analysis

Soil samples were obtained by composite sampling at each site. Soil pH was determined in a 1:5 (w/v) soil-to-water suspension using a calibrated pH meter, and EC_e_ was measured with a conductivity meter. Soil organic carbon (SOC) was analyzed using the Walkley–Black method (Walkley & Black, 1934), and total organic nitrogen (TON) was determined by Kjeldahl digestion (Bremner, 1960). Exchangeable cations (K^+^, Na^+^, Ca^2^^+^, and Mg^2^^+^) were extracted with 1 N ammonium acetate (pH 7.0) following the Land Development Department (2010) protocol and quantified by atomic absorption spectroscopy (AAS; Agilent 280FS AA). Chloride (Cl^−^) concentration was determined by Mohr titration (Sheen & Kahler, 1938), while Fe and Zn concentrations were measured by AAS. The SAR was calculated as:

\begin{eqnarray*}SAR= \frac{N{a}^{+}}{\sqrt{(C{a}^{2+}+M{g}^{2+})/2}} \end{eqnarray*}

with all concentrations expressed in cmolc kg^−^^1^.

### Extraction of RBO

RBO was extracted using a Soxhlet apparatus with n-hexane at a bran-to-solvent ratio of 1:20 (w/v) for 72 h to ensure exhaustive oil recovery. The 72 h extraction period was selected to maximize exhaustive oil recovery across all field samples and was applied consistently to all treatments to allow comparative analysis. The solvent was removed under reduced pressure with a rotary evaporator, and the crude oil yield was calculated gravimetrically. Extracts were stored in amber glass bottles at 4 °C until analysis.

### γ-oryzanol determination

Total γ-oryzanol in RBO was quantified by UV–Vis spectrophotometry following the method of Bucci et al. (2003), with slight modifications. An aliquot of approximately 0.10 g of oil was diluted to 25 mL with isopropanol in a volumetric flask and mixed thoroughly. The absorbance of the solution was measured at 327 nm using a UV–Vis spectrophotometer with isopropanol as the blank. Calibration was performed using commercial γ-oryzanol standards prepared in isopropanol at known concentrations. The concentration of γ-oryzanol in the samples was calculated from the standard curve and expressed as grams per 100 g of oil (g 100 g^−^^1^).

### Determination of total carotenoids

Total carotenoids in RBO were determined spectrophotometrically following the AOAC method (AOAC, 1958; AOAC, 1973), with modifications for oil samples. Approximately 1.0 g of crude oil was dissolved in 25 mL of n-hexane in a volumetric flask. The absorbance of the solution was measured at 450 nm using a UV–Vis spectrophotometer (hexane as blank). Carotenoid content was calculated and expressed as milligrams of β-carotene equivalents per kilogram of oil (mg β-carotene eq. kg^−^^1^), according to the following equation:

\begin{eqnarray*}Carotenoids= \frac{A\times 1{0}^{6}}{{E}_{1cm}^{1\%}\times c} \end{eqnarray*}

where *A* is the absorbance at 450 nm, *c* is the concentration of oil solution (g 100 mL^−^^1^), and *E*${}_{\mathrm{1cm}}^{1\%}$ is the extinction coefficient for β-carotene, 2,592 at 450 nm.

### Determination of total phenolic content

Total phenolic content was determined in accordance with the method described by Trevisani Juchen et al. (2019). Briefly, 50 mg of RBO was mixed with one mL of methanol. An aliquot of 0.5 mL of this extract was combined with 2.5 mL of Folin–Ciocalteu reagent and shaken for 6 min, followed by the addition of two mL of 10% (w/v) sodium carbonate solution. The mixture was incubated at room temperature in the dark for 2 h. Absorbance was measured at 765 nm using a UV–Vis spectrophotometer. Results were expressed as micrograms of gallic acid equivalent per gram of oil (µg GAE g^−^^1^).

### Determination of total flavonoid content

Total flavonoid content was analyzed according to the method of Jun et al. (2012). An aliquot of 0.5 mL of RBO was dissolved in 4.5 mL of methanol. The solution was mixed with 1.25 mL distilled water and 75 µL of 5% NaNO_2_ and incubated at room temperature for 6 min. Then, 150 µL of 10% AlCl_3_ solution was added and kept in the dark for 5 min, followed by 0.5 mL of 1.0 M NaOH. The mixture was shaken vigorously, and absorbance was measured at 510 nm using a UV–Vis spectrophotometer. Catechin was used as a standard, and results were expressed as micrograms catechin equivalent per gram of oil (µg CE g^−^^1^).

### Determination of antioxidant activity

The antioxidant activity of RBO was determined using the 2,2-diphenyl-1-picrylhydrazyl (DPPH) free radical scavenging method, following Trevisani Juchen et al. (2019). A stock solution of DPPH (0.1 mM) in methanol was prepared. An aliquot of 0.1 mL of oil extract (dissolved in methanol at appropriate concentrations) was added to 3.9 mL of the DPPH solution, vortexed, and incubated in the dark at room temperature for 30 min. Absorbance was measured at 517 nm using a UV–Vis spectrophotometer. Trolox was used as the reference antioxidant standard, and results were expressed as milligrams Trolox equivalent antioxidant capacity per gram of oil (mg TEAC g^−^^1^).

### Determination of fatty acid profile

Fatty acid composition was determined by gas chromatography–mass spectrometry (GC–MS) after conversion of RBO to fatty acid methyl esters (FAMEs) using methanolic NaOH and BF_3_–MeOH, with C17:0 as an internal standard. Extracted FAMEs were analyzed on an Agilent 7890A GC coupled to an Agilent 7000B MS, equipped with a DB-Wax capillary column (60 m × 0.25 mm, 0.25 µm). Helium was used as the carrier gas at a flow rate of 1.0 mL/min. Injections (1 µL) were performed at 240 °C in split mode (100:1). The oven temperature program was 70 °C (4 min), ramped at 13 °C/min to 175 °C (27 min), and then at 3 °C/min to 240 °C (30 min). The MS operated in EI mode (70 eV; scan range m/z 35–550) at an ion source temperature of 230 °C. Fatty acids were identified using authentic standards and the NIST library, and results were expressed as relative percentages of total FAMEs.

### Determination of elements in RBO

Elements (K, Na, Ca, Fe, Mg, and Zn) in RBO were determined after acid digestion. Approximately 2 g of oil was digested with concentrated nitric acid until a clear solution was obtained, then diluted with deionized water. Elemental concentrations were quantified using atomic absorption spectroscopy (AAS; Agilent 280FS AA) and expressed as mg kg^−^^1^ of oil.

### Synchrotron radiation-based FTIR analysis

Four representative RBO samples from contrasting soil salinity levels, together with a commercial RBO reference, were analyzed using synchrotron radiation–based Fourier transform infrared microspectroscopy (SR-FTIR). Measurements were performed in reflection mode at Beamline 4.1 (IR Spectroscopy and Imaging), Synchrotron Light Research Institute (SLRI), Thailand, using a Vertex 70 FTIR spectrometer coupled with a Hyperion 2000 infrared microscope equipped with 36 × Cassegrain objectives and a liquid-nitrogen-cooled MCT detector (Bruker Corporation, Ettlingen, Germany), a setup comparable to recently reported synchrotron infrared beamline facilities (Chio-Srichan et al., 2026).

Approximately 2–3 drops of oil were deposited directly onto MirrIR low-e reflective microscope slides (Keiley Technologies, USA). Spectra were collected over the mid-infrared range of 4,000–600 cm^−^^1^ at a spectral resolution of four cm^−^^1^ with 64 co-added scans. All spectra were baseline-corrected and normalized using OPUS 7.8 software (Bruker Optics, Ettlingen, Germany). Spectral interpretation focused on characteristic absorption bands of triacylglycerol, including C–H stretching vibrations (3,000–2,800 cm^−^^1^), ester carbonyl stretching (1,750–1,740 cm^−^^1^), and C–O/C–O–C vibrations in the fingerprint region (1,500–950 cm^−^^1^) (Miglio et al., 2013).

### Statistical analysis

All measurements were conducted in analytical triplicate and averaged at the field-site level prior to analysis. Results are expressed as mean ± SE, except for fatty acid data, which are reported as mean ± SD. Field sites were treated as biological replicates, with unequal sample sizes across salinity × variety groups (generally n = 3–4 per group; total *n* = 14). Fatty acid composition was analyzed on a subset of samples, as indicated in Table 1. The effects of salinity level, rice variety, and their interaction were evaluated using two-way ANOVA with Type III sums of squares. Effect sizes for two-way ANOVA were estimated using partial eta squared (*η*p^2^). Normality of residuals and homogeneity of variance were assessed using Shapiro–Wilk and Levene’s tests, respectively. For graphical presentation, pairwise comparisons among salinity × variety combinations were further examined using Tukey’s honestly significant difference (HSD) test where appropriate, and different lowercase letters indicate significant differences among treatment means at *p* < 0.05. Principal component analysis (PCA) was used as an exploratory tool to visualize multivariate relationships, supported by univariate ANOVA and Pearson correlation analyses. All analyses were performed using IBM SPSS Statistics v29.0.0.0 (IBM Corp., Armonk, NY, USA), and statistical significance was set at *p* < 0.05.

**Table 1:** Fatty acid composition (% of total fatty acids) of rice bran oil extracted from RD6 and KDML105 cultivated under low- and high-salinity soil conditions.

Fatty acid composition	Low salinity		High salinity		*p*-value (Type III ANOVA)		
	RD6	KDML105	RD6	KDML105	Salinity (S)	Variety (V)	S × V
**Saturated fatty acids (SFA)**							
Myristic acid (C14:0)	0.28 ± 0.04	0.32 ± 0.08	0.35 ± 0.06	0.33 ± 0.08	0.372	0.866	0.591
Palmitic acid (C16:0)	17.54 ± 0.12	17.84 ± 5.10	14.55 ± 7.20	8.53 ± 0.41	0.111	0.418	0.375
Stearic acid (C18:0)	2.14 ± 0.04	2.20 ± 0.23	1.74 ± 0.46	1.39 ± 0.06	0.019^*^	0.487	0.319
Arachidic acid (C20:0)	0.77 ± 0.02	0.79 ± 0.17	0.53 ± 0.14	0.42 ± 0.05	0.011^*^	0.573	0.473
Behenic acid (C22:0)	0.30 ± 0.06	0.30 ± 0.07	0.22 ± 0.09	0.17 ± 0.03	0.057	0.559	0.589
Lignoceric acid (C24:0)	0.48 ± 0.09	0.48 ± 0.10	0.36 ± 0.21	0.24 ± 0.05	0.098	0.523	0.506
**Monounsaturated fatty acids (MUFA)**							
Palmitoleic acid (C16:1)	0.26 ± 0.00	0.27 ± 0.03	0.26 ± 0.07	0.31 ± 0.02	0.448	0.396	0.437
Oleic acid (C18:1)	44.65 ± 0.29	44.22 ± 4.43	44.61 ± 4.25	49.24 ± 0.86	0.321	0.396	0.313
cis-11-Eicosenoic acid (C20:1)	0.49 ± 0.02	0.46 ± 0.06	0.42 ± 0.06	0.52 ± 0.08	0.869	0.371	0.175
**Polyunsaturated fatty acids (PUFA)**							
Linoleic acid (C18:2n6c)	31.67 ± 0.28	31.75 ± 2.39	35.57 ± 3.51	37.26 ± 1.57	0.028^*^	0.609	0.643
α-Linolenic acid (C18:3n3)	1.42 ± 0.02	1.38 ± 0.04	1.39 ± 0.19	1.60 ± 0.13	0.276	0.304	0.155
**Summary**							
ΣSFA	21.51 ± 0.03	21.92 ± 5.58	17.75 ± 8.03	11.08 ± 0.52	0.092	0.423	0.369
ΣMUFA	45.40 ± 0.27	44.95 ± 4.49	45.29 ± 4.37	50.07 ± 0.92	0.327	0.393	0.309
ΣPUFA	33.09 ± 0.30	33.13 ± 2.42	36.96 ± 3.69	38.86 ± 1.44	0.030^*^	0.586	0.604
MUFA/SFA	2.11 ± 0.01	2.17 ± 0.72	2.95 ± 1.32	4.52 ± 0.13	0.030^*^	0.196	0.227
PUFA/SFA	1.54 ± 0.02	1.58 ± 0.43	2.41 ± 1.10	3.52 ± 0.29	0.021^*^	0.245	0.281
UFA/SFA	3.65 ± 0.01	3.75 ± 1.14	5.35 ± 2.42	8.04 ± 0.42	0.025^*^	0.213	0.247

**Notes.**

Values are presented as mean ± SD (% of total fatty acids), with field sites treated as biological replicates (*n* = 2–3 per salinity × variety group). Differences were evaluated by two-way ANOVA using Type III sums of squares to account for the unbalanced design. Exact *p*-values are reported for the main effects of salinity (S), variety (V), and their interaction (S × V). No significant S × V interactions were detected for any fatty acid variable.

* *p* < 0.05.

## Results

### Soil salinity classification and soil chemical characteristics

**Figure 2:**
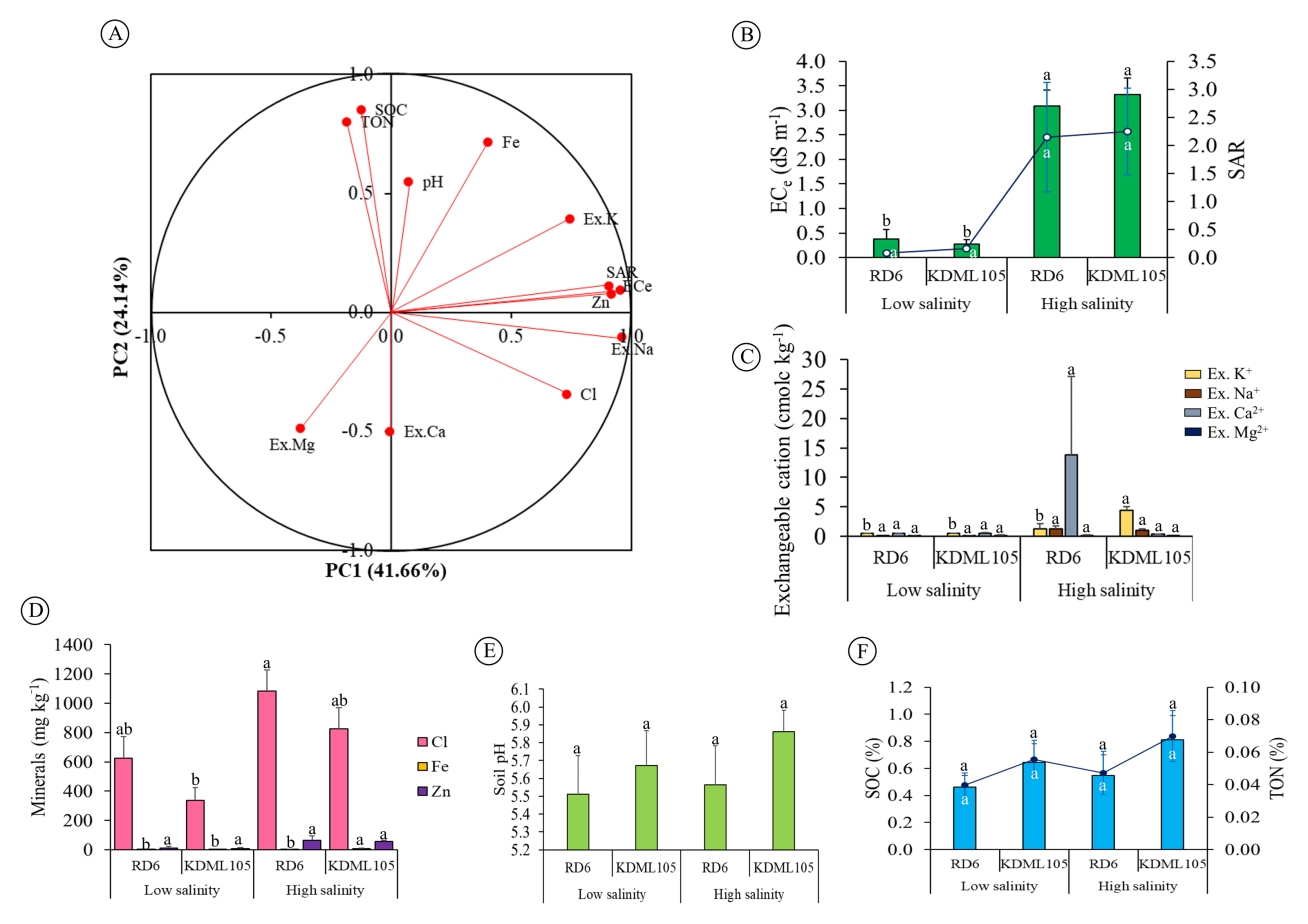
Soil chemical properties and multivariate patterns under contrasting salinity conditions. (A) PCA biplot of soil chemical variables; percentages indicate the variance explained by each principal component. (B) EC_e_ (bars, left axis) and SAR (line, right axis). (C) Exchangeable cations (K^+^, Na^+^, Ca^2^^+^, and Mg^2^^+^). (D) Selected soil minerals (Cl^-^, Fe, and Zn). (E) Soil pH. (F) SOC (bars, left axis) and TON (line, right axis). Values are means ± SE (n = 3–4 per group). Different lowercase letters indicate significant differences among salinity × variety group means within each variable based on Tukey’s HSD test at *p* < 0.05.

Soils were categorized into low- and high-salinity classes based on EC_e_ thresholds, enabling comparative assessment of soil chemical variability (Fig. 2). PCA of soil variables explained 65.80% of the total variance, with PC1 (41.66%) representing salinity-related gradients and PC2 (24.14%) reflecting secondary variation associated with soil fertility-related attributes (Fig. 2A). Salinity indicators, including EC_e_, SAR, Cl^−^, and exchangeable Na^+^ and K^+^, loaded positively on PC1, whereas SOC, TON, pH, soil Fe, and exchangeable Ca^2^^+^ and Mg^2^^+^ were more strongly associated with PC2.

Consistent with these patterns, two-way ANOVA showed significant salinity effects on EC_e_ (F_1_,_1__0_ = 126.97, *p* < 0.001, *ηp*^2^ = 0.927), SAR (F_1_,_1__0_ = 9.82, *p* = 0.011, *ηp*^2^ = 0.496), soil Cl^−^ (F_1_,_1__0_ = 12.47, *p* = 0.005, *ηp*^2^ = 0.555), soil Fe (F_1_,_1__0_ = 12.77, *p* = 0.005, *ηp*^2^ = 0.561), soil Zn (F_1_,_1__0_ = 7.32, *p* = 0.022, *ηp*^2^ = 0.423), exchangeable Na^+^ (F_1_,_1__0_ = 12.08, *p* = 0.006, *ηp*^2^ = 0.547), and exchangeable K^+^ (F_1_,_1__0_ = 20.05, *p* = 0.001, *ηp*^2^ = 0.667) (Figs. 2B–2D). Soil Fe was also significantly affected by rice variety (F_1_,_1__0_ = 6.41, *p* = 0.030, *ηp*^2^ = 0.391). Exchangeable K^+^ was likewise affected by rice variety (F_1_,_1__0_ = 8.48, *p* = 0.016, *ηp*^2^ = 0.459), and a significant salinity × variety interaction was detected (F_1_,_1__0_ = 8.46, *p* = 0.016, *ηp*^2^ = 0.458), indicating that the response of exchangeable K^+^ to salinity differed between RD6 and KDML105. In contrast, soil pH, SOC, TON, and exchangeable Ca^2^^+^ and Mg^2^^+^ were not significantly affected by salinity, variety, or their interaction (Figs. 2C, 2E, 2F).

### RBO properties and antioxidant capacity under contrasting soil salinity levels

**Figure 3:**
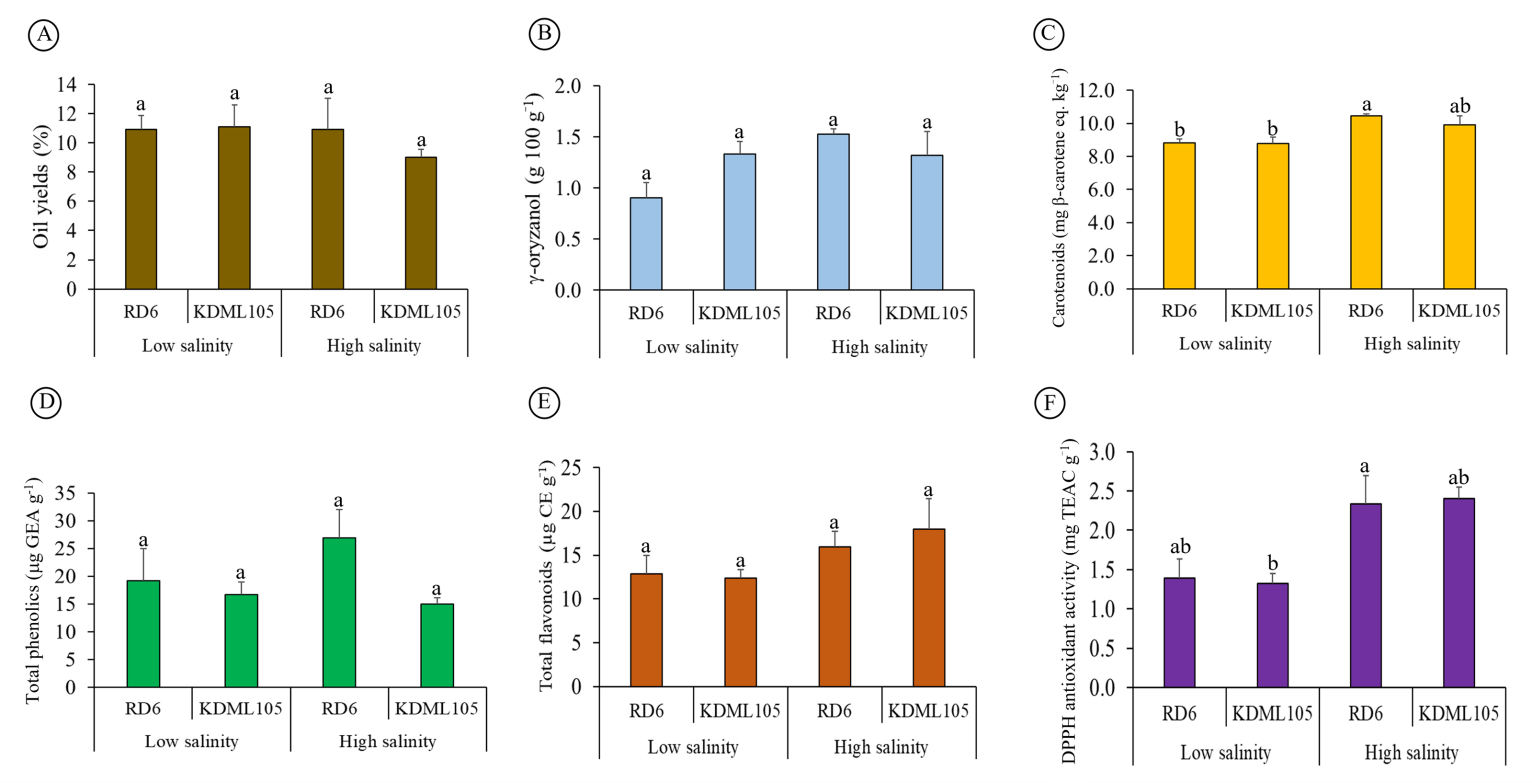
Oil yield, bioactive compounds, and antioxidant capacity of rice bran oil under contrasting soil salinity conditions. (A) Oil yield. (B) γ-oryzanol content. (C) Total carotenoids, expressed as β-carotene equivalents. (D) Total phenolic content, expressed as gallic acid equivalents (GAE). (E) Total flavonoid content, expressed as catechin equivalents (CE). (F) DPPH antioxidant activity, expressed as Trolox equivalent antioxidant capacity (TEAC). Values are means  ± SE (n = 3–4 per group). Different lowercase letters indicate significant differences among salinity × variety group means within each variable based on Tukey’s HSD test at *p* < 0.05.

RBO yield ranged from approximately 8–12% and did not differ significantly with salinity, rice variety, or their interaction (*p* > 0.05), indicating relative stability of oil yield across field conditions (Fig. 3A). In contrast, γ-oryzanol content was significantly influenced by the salinity × variety interaction (F_1_,_1__0_ = 5.11, *p* = 0.047, *ηp*^2^ = 0.338), whereas the salinity and variety main effects were not significant (F_1_,_1__0_ = 4.69, *p* = 0.056 and F_1_,_1__0_ = 0.59, *p* = 0.460, respectively), indicating that the response of γ-oryzanol to salinity differed between RD6 and KDML105 (Fig. 3B).

Total carotenoid content was significantly higher under high-salinity conditions (F_1_,_1__0_ = 15.91, *p* = 0.003, *ηp*^2^ = 0.614), with no significant effects of rice variety or the salinity × variety interaction (Fig. 3C). Total phenolic and total flavonoid contents showed no significant effects of salinity, variety, or the interaction term (*p* > 0.05) (Figs. 3D, 3E). Antioxidant activity, assessed by DPPH radical scavenging capacity, was significantly higher under high salinity (F_1_,_1__0_ = 16.12, *p* = 0.002, *ηp*^2^ = 0.617), whereas rice variety and the salinity × variety interaction were not significant (Fig. 3F).

### Fatty acid composition of RBO

Across all treatments, RBO was dominated by palmitic acid (C16:0) among saturated fatty acids (SFA), oleic acid (C18:1) among monounsaturated fatty acids (MUFA), and linoleic acid (C18:2n6c) among polyunsaturated fatty acids (PUFA) (Table 1). Salinity was significantly associated with variation in several fatty acid variables. Among the individual fatty acids, stearic acid (C18:0), arachidic acid (C20:0), and linoleic acid (C18:2n6c) showed significant salinity effects (F_1_,_6_ = 10.11, *p* = 0.019, *ηp*^2^ = 0.628; F_1_,_6_ = 13.31, *p* = 0.011, *ηp*^2^ = 0.689; and F_1_,_6_ = 8.27, *p* = 0.028, *ηp*^2^ = 0.579, respectively). Stearic and arachidic acids were lower under high-salinity conditions, whereas linoleic acid was higher under high salinity. In contrast, myristic acid (C14:0), palmitic acid (C16:0), behenic acid (C22:0), lignoceric acid (C24:0), palmitoleic acid (C16:1), oleic acid (C18:1), cis-11-eicosenoic acid (C20:1), and α-linolenic acid (C18:3n3) were not significantly affected by salinity (*p* > 0.05).

When grouped variables were considered, Σ PUFA was significantly higher under high salinity (F_1_,_6_ = 8.07, *p* = 0.030, *ηp*^2^ = 0.573), whereas Σ SFA and Σ MUFA were not significantly affected (*p* > 0.05). In addition, the MUFA/SFA, PUFA/SFA, and UFA/SFA ratios were significantly higher under high-salinity conditions (F_1_,_6_ = 8.04, *p* = 0.030, *ηp*^2^ = 0.573; F_1_,_6_ = 9.73, *p* = 0.021, *ηp*^2^ = 0.618; and F_1_,_6_ = 8.90, *p* = 0.025, *ηp*^2^ = 0.597, respectively), indicating a shift toward a relatively more unsaturated fatty acid profile under saline conditions. No significant effects of rice variety or salinity × variety interaction were detected for any fatty acid variable.

### Elemental composition of RBO under contrasting soil salinity levels

**Figure 4:**
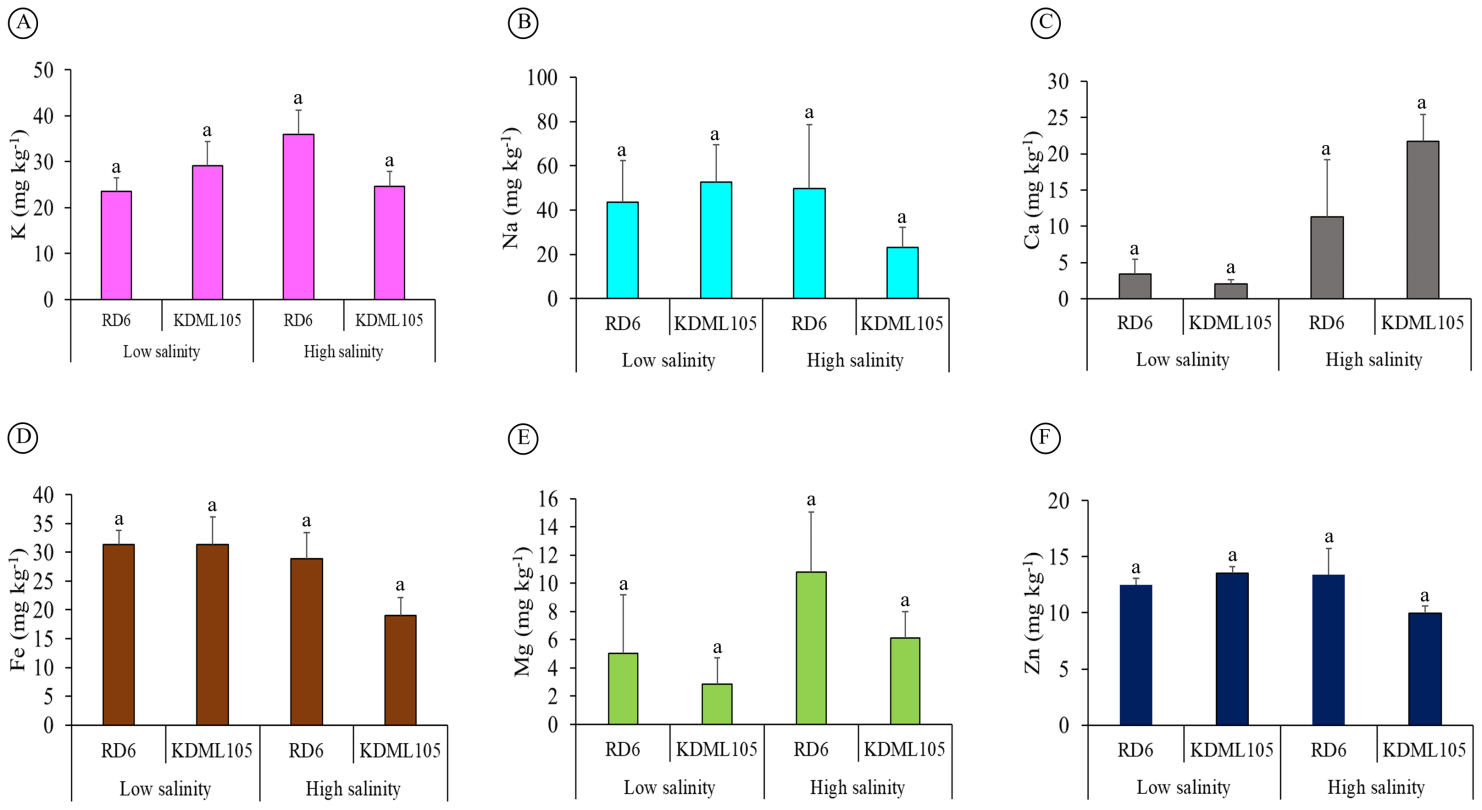
Elemental composition of rice bran oil under contrasting soil salinity conditions. (A) Potassium (K), (B) sodium (Na), (C) calcium (Ca), (D) iron (Fe), (E) magnesium (Mg), and (F) zinc (Zn) concentrations in rice bran oil (RBO). Values are means ± SE (n = 3–4 per group). Different lowercase letters, where shown, indicate significant differences among salinity × variety group means within each variable based on Tukey’s HSD test at *p* < 0.05.

The elemental composition of RBO showed limited responses to soil salinity (Fig. 4). Among the measured elements, only calcium (Ca) was significantly affected by salinity, with higher concentrations in RBO extracted from rice grown under high-salinity conditions (F_1_,_1__0_ = 7.59, *p* = 0.020, *ηp*^2^ = 0.431) (Fig. 4C). In contrast, the concentrations of K, Na, Fe, Mg, and Zn were not significantly affected by salinity, rice variety, or the salinity × variety interaction (*p* > 0.05) (Figs. 4A, 4B, 4D–4F).

### SR-FTIR spectral characteristics of RBO

**Figure 5:**
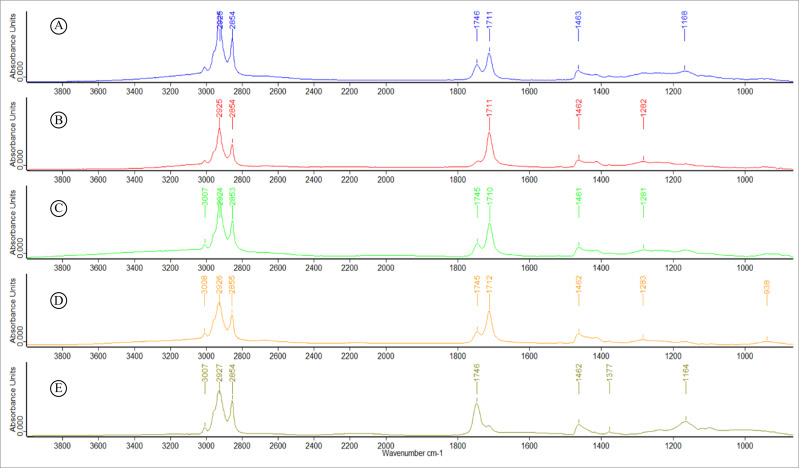
Synchrotron FTIR spectra of rice bran oil. Low-salinity samples are shown in panels (A, B) (RD6, G5 site; KDML105, N7 site), high-salinity samples in panels (C, D) (RD6, G4 site; KDML105, N4 site), and a commercial rice bran oil (RBO) reference in panel (E). All spectra display characteristic triacylglycerol absorption bands, including =C–H stretching (∼3,007 cm^−1^), CH_2_/CH_3_ stretching (∼2,923 and ∼2,853 cm^−1^), ester carbonyl (C=O) stretching (∼1,745 cm^−1^), CH_2_ and CH_3_ bending vibrations (∼1,460 and ∼1,375 cm^−1^), and C–O/C–O–C stretching vibrations in the fingerprint region (∼1,238–1,114 cm^−1^).

SR-FTIR spectra of representative RBO samples from low- and high-salinity conditions, together with a commercial RBO reference, exhibited highly similar absorption patterns characteristic of triacylglycerol (Fig. 5). All spectra showed prominent bands corresponding to =C–H stretching vibrations (∼3,007 cm^−^^1^), CH_2_ and CH_3_ stretching (∼2,923 and ∼2,853 cm^−^^1^), and ester carbonyl (C=O) stretching (∼1,745 cm^−^^1^). Additional absorption bands associated with CH_2_ and CH_3_ bending vibrations were observed at approximately 1,460 and 1,375 cm^−^^1^, while C–O and C–O–C stretching vibrations appeared in the fingerprint region (1,238–1,114 cm^−^^1^).

No distinct peak shifts, intensity anomalies, or additional functional groups were detected among samples, indicating that soil salinity influenced RBO composition quantitatively rather than altering the fundamental triacylglycerol structure, regardless of rice variety.

### Multivariate relationships among soil properties and RBO traits

**Figure 6:**
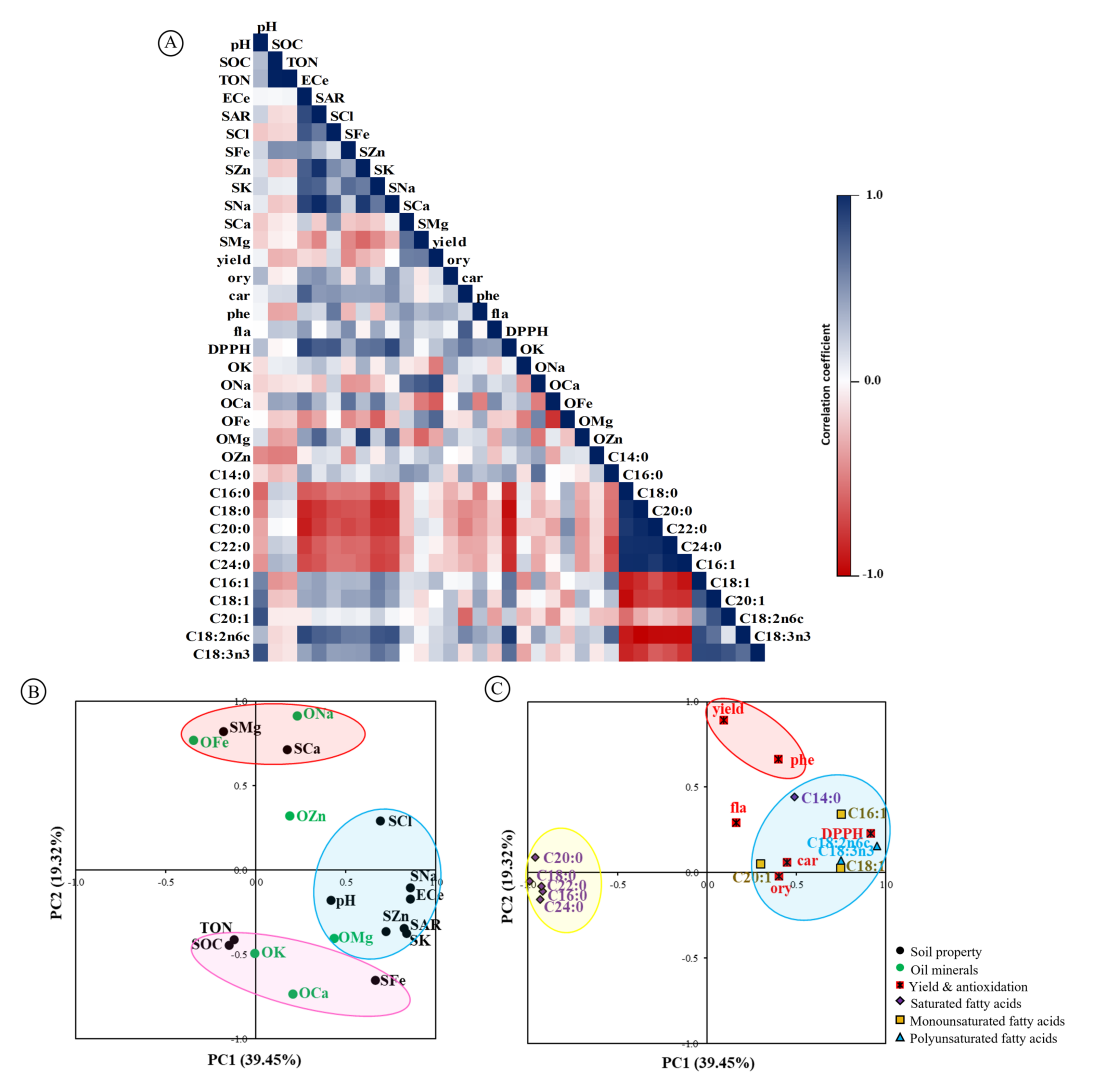
Correlation and principal component analysis (PCA) of soil properties and rice bran oil traits. (A) Pearson correlation matrix of soil chemical variables, RBO yield, bioactive components, elemental composition, and fatty acid profiles; color intensity indicates the strength and direction of correlations (blue, positive; red, negative). (B, C) PCA biplot from a single analysis, presented in two panels for clarity: (B) soil chemical properties and soil elemental variables; (C) RBO yield, antioxidant-related traits, and individual fatty acids and fatty acid classes. Percentages indicate the variance explained by PC1 and PC2. Abbreviations: SK, SNa, SCa, and SMg, exchangeable K^+^, Na^+^, Ca^2^^+^, and Mg^2+^ in soil; SCl, SFe, and SZn, soil Cl^-^, Fe, and Zn; OK, ONa, OCa, OFe, OMg, and OZn, K, Na, Ca, Fe, Mg, and Zn in oil; phe, total phenolics; fla, total flavonoids; ory, γ-oryzanol; car, total carotenoids; DPPH, antioxidant activity.

PCA integrating soil properties and RBO traits explained 58.77% of the total variance, with PC1 (39.45%) and PC2 (19.32%) capturing the major gradients (Figs. 6B, 6C). PC1 represented a dominant salinity–oil quality gradient, with positive loadings for salinity-related soil variables (EC_e_, SAR, exchangeable Na^+^ and K^+^, Cl^−^, and Zn), antioxidant activity (DPPH), and unsaturated fatty acids (C16:1, C18:1, C18:2n6c, and C18:3n3), while saturated fatty acids—except C14:0—loaded negatively.

PC2 reflected a secondary gradient associated with yield and elemental variation, with positive loadings for yield, soil Ca^2^^+^ and Mg^2^^+^, oil Na and Fe, and total phenolics, and negative loadings for soil Fe, oil Ca and K, and, to a lesser extent, SOC and TON. Overall, the PCA indicates that salinity-related soil conditions align with greater lipid unsaturation and antioxidant activity (PC1), whereas yield and selected elemental patterns vary independently along PC2.

Pearson correlation analysis supported these patterns (Fig. 6A), showing positive associations between salinity indicators, unsaturated fatty acids, and antioxidant activity, and negative associations with saturated fatty acids. Oil yield exhibited weak or inconsistent correlations with salinity indicators and fatty acid composition along the primary salinity–lipid axis.

## Discussion

Soil salinity was strongly associated with variation in the compositional quality of RBO in both RD6 and KDML105 cultivated along the Siao Yai River. Multivariate analyses showed that salinity-related soil variables, particularly EC_e_, SAR, chloride, and exchangeable Na^+^ and K^+^, were closely associated with variation in fatty acid composition and antioxidant activity, indicating a major salinity-related gradient in RBO quality. This interpretation was consistent with the univariate analysis, which showed significant salinity effects on exchangeable Na^+^, exchangeable K^+^, chloride, Fe, and Zn. In contrast, SOC and TON were aligned along a secondary PCA axis together with yield and selected elemental variables, indicating that fertility- and yield-related factors varied independently of the primary salinity-driven gradient. The clear orthogonal separation of salinity-associated variables from fertility-, yield-, and element-related patterns in PCA supports the interpretation that salinity was more closely associated with RBO compositional variation than other measured soil properties under the present field conditions.

### Salinity-induced lipid desaturation and fatty acid restructuring

Building on the multivariate separation observed between salinity and fertility variables, fatty acid composition was among the oil quality traits associated with soil salinity. The opposing alignment of EC_e_ and SAR with saturated fatty acids, together with their positive association with unsaturated fatty acids, including both MUFA such as oleic acid and PUFA such as linoleic acid (C18:2n6c), suggests that saline environments were associated with a shift toward greater lipid unsaturation in rice grain oils. This response is consistent with current understanding of plant salt-stress physiology, in which membrane lipid remodeling and increased fatty acid unsaturation contribute to membrane homeostasis and stress acclimation under saline conditions (Guo et al., 2022; Pan et al., 2023). Similar patterns have been reported in rice grains exposed to salinity, where enzymatic desaturation converts saturated fatty acids into mono- and polyunsaturated forms as part of stress adaptation (Razzaq et al., 2020; Li et al., 2023).

In addition to the multivariate pattern, the univariate analysis showed significant salinity effects for stearic (C18:0), arachidic acid (C20:0), and linoleic acid (C18:2n6c), while Σ PUFA and the MUFA/SFA, PUFA/SFA, and UFA/SFA ratios were also significantly higher under high-salinity conditions. By contrast, Σ SFA, Σ MUFA, and several major individual fatty acids, including oleic acid, were not significantly affected. Taken together, these results suggest that the salinity-associated improvement in fatty acid quality was expressed more clearly through reduced proportions of specific saturated fatty acids and increased unsaturated-to-saturated fatty acid ratios than through uniform changes across all fatty acid classes. From a nutritional and technological perspective, a shift toward a relatively more unsaturated fatty acid profile—including higher unsaturated-to-saturated fatty acid ratios and greater linoleic acid proportion under high salinity—is favorable, as it supports cardiovascular health and enhances the functional quality of edible oils (Most et al., 2005; Xu et al., 2021).

### Antioxidant capacity and bioactive constituents under salinity stress

In parallel with changes in fatty acid composition, salinity also influenced the antioxidant-related properties of RBO. Antioxidant activity, measured by DPPH radical scavenging capacity, increased significantly under high salinity and closely aligned with unsaturated fatty acids in multivariate space. Such enhancement of antioxidant capacity under saline conditions is consistent with evidence that moderate salt stress activates antioxidant defense systems in plants, often increasing the accumulation and activity of both enzymatic and non-enzymatic antioxidants (Azeem et al., 2023). This pattern suggests that lipid compositional variation may contribute to enhanced antioxidant capacity in RBO.

Among lipid-associated antioxidants, γ-oryzanol content showed a variety-dependent response to salinity, with a significant salinity × variety interaction, whereas correlation analysis showed that antioxidant activity was more strongly associated with salinity indicators and unsaturated fatty acids than with γ-oryzanol alone, suggesting a synergistic rather than dominant role of γ-oryzanol in determining antioxidant activity (Zhao et al., 2021). Total carotenoid content also increased with salinity, indicating activation of antioxidant-related metabolic pathways under stress. Carotenoids play a key role in quenching reactive oxygen species and protecting cellular structures, and their accumulation under saline conditions is consistent with stress-induced antioxidant responses observed in other plant systems (Pungin et al., 2023).

In contrast, total phenolic and flavonoid contents were not significantly affected by salinity, indicating a more limited contribution of hydrophilic phenolic compounds to the observed antioxidant response. Together, these results suggest that antioxidant enhancement in RBO under salinity is driven primarily by lipid-associated antioxidants and unsaturated fatty acids rather than by phenolic accumulation.

### Oil yield and separation of quantity *versus* quality responses

Notably, RBO yield was not significantly affected by soil salinity and was clearly separated from lipid compositional and antioxidant traits in PCA space. This suggests that salinity was associated with oil quality rather than oil quantity under field conditions. Such decoupling suggests that rice plants maintain oil accumulation while reallocating metabolic fluxes toward stress-adaptive lipid profiles, an observation with important agronomic and processing implications.

### Elemental composition and soil–oil linkages

Elemental composition of RBO showed limited responses to salinity, with calcium (Ca) being the only measured element that differed significantly under high-salinity conditions. This response suggests that soil ionic conditions may contribute to Ca accumulation in RBO, even though Ca did not consistently co-vary with salinity indicators across all sites in multivariate space. In contrast, K, Na, Mg, Fe, and Zn exhibited greater site-specific variability and were not significantly affected by salinity or variety. While previous studies have emphasized extraction methods or genetic variation as primary drivers of RBO elemental composition (Xu et al., 2021; Wisetkomolmat et al., 2022), the present findings suggest that edaphic conditions may also contribute to variation in selected oil elemental traits under field conditions. Because mineral composition contributes to nutritional quality, these results provide additional insight into how saline soils may influence rice-derived food products (Oo et al., 2025).

### Structural stability of triacylglycerols under salinity

SR-FTIR analysis of representative RBO samples demonstrated that triacylglycerol structures were preserved regardless of salinity level or rice variety. The absence of peak shifts, new functional groups, or hydrolysis-related features indicates that salinity-driven effects are compositional rather than structural. This structural stability is consistent with plant stress responses in which fatty acid composition is adjusted without alteration of the ester backbone of storage lipids (Miglio et al., 2013).

### Implications for RBO valorization in saline-prone systems

Importantly, the present field-based results suggest that salinity-associated improvements in RBO quality may occur under realistic agronomic conditions, extending previous controlled-environment studies to on-farm production systems.

From an applied perspective, enrichment of unsaturated fatty acids and lipid-associated antioxidants highlights the potential value of RBO produced in saline-prone agricultural regions. Because extraction and refining processes influence bioactive retention and sterol stability (Mathias et al., 2023; Yu et al., 2023; Pimpa, Thongraung & Sutthirak, 2021), optimized processing may further enhance the benefits of salinity-associated RBO. Emerging applications, such as RBO-based oleogels developed as trans-fat-free solid fat alternatives, underscore the expanding role of RBO in food and material applications (Sivakanthan et al., 2023). Collectively, these findings position saline environments not only as constraints but also as opportunities for producing nutritionally enhanced RBO.

The enrichment of lipid-associated antioxidants observed under saline field conditions is consistent with reports demonstrating that rice bran–derived components enhance oxidative stability when incorporated into edible oil systems (Olagunju, Adelakun & Olawoyin, 2022).

## Conclusions

Soil salinity was strongly associated with the compositional quality and functional attributes of RBO derived from both RD6 and KDML105 cultivated along the Siao Yai River. Elevated EC_e_ and SAR were associated with changes in fatty acid composition, including significant reductions in specific saturated fatty acids (C18:0 and C20:0), higher linoleic acid (C18:2n6c), and increases in unsaturated-to-saturated fatty acid ratios, indicating a shift toward a relatively more unsaturated lipid profile under saline conditions. Antioxidant capacity increased with salinity and was closely aligned with salinity indicators and unsaturated fatty acids, alongside higher carotenoid contents and a variety-dependent response of γ-oryzanol to salinity. In contrast, RBO yield remained unaffected by salinity, indicating that soil salinity altered oil quality rather than oil quantity under field conditions. Elemental composition of RBO showed limited responses to edaphic conditions, with calcium enrichment under high salinity, while SR-FTIR analysis confirmed that salinity-associated changes occurred without alteration of fundamental triacylglycerol structures. Overall, the results suggest that saline environments may enhance the nutritional and functional quality of RBO while maintaining yield, supporting the potential valorization of RBO produced in saline-prone agricultural systems.

##  Supplemental Information

10.7717/peerj.21476/supp-1 Supplemental Information 1 Site codes and raw soil chemical properties of rice cultivation sites Site codes, geographic information, rice cultivar identity, salinity classification, and raw soil chemical properties measured at rice cultivation sites along the Siao Yai River basin, northeastern Thailand.
Soil parameters include electrical conductivity of the saturated paste extract (EC_e_), sodium adsorption ratio (SAR), soil pH, soil organic carbon (SOC), total organic nitrogen (TON), chloride (Cl^-^), exchangeable cations (K^+^, Na^+^, Ca^2+^, and Mg^2+^), and selected soil elemental concentrations.
These data support the spatial distribution of sampling sites shown in Figure 1 and the soil chemical comparisons and multivariate analyses presented in Figure 2. The dataset was used for statistical analyses including two-way analysis of variance (ANOVA), correlation analysis, and principal component analysis (PCA).

10.7717/peerj.21476/supp-2 Supplemental Information 2 Raw data for rice bran oil yield, bioactive compounds, and antioxidant activity Raw data for rice bran oil (RBO) yield and bioactive-related properties measured in RD6 and KDML105 cultivated under low- and high-salinity conditions. Variables include oil yield, γ-oryzanol content, total carotenoids, total phenolic content, total flavonoid content, and DPPH radical scavenging activity. Data are reported at the field-site level and were used for statistical analyses presented in Figure 3 and associated Results sections.

10.7717/peerj.21476/supp-3 Supplemental Information 3 Raw fatty acid composition data of rice bran oil Raw fatty acid composition data of rice bran oil (RBO) extracted from RD6 and KDML105 cultivars grown under low- and high-salinity conditions. Individual fatty acids are reported as relative percentages of total fatty acid methyl esters (FAMEs), as determined by GC–MS.
These raw data were used for statistical analyses and for generating the summarized results presented in Table 1 and Figure 6.

10.7717/peerj.21476/supp-4 Supplemental Information 4 Raw elemental composition data of rice bran oil Raw elemental composition data of rice bran oil (RBO) extracted from RD6 and KDML105 cultivars grown under low- and high-salinity conditions. Elements quantified include potassium (K), sodium (Na), calcium (Ca), iron (Fe), magnesium (Mg), and zinc (Zn).
Data are reported at the field-site level and were used for statistical analyses and for generating Figure 4 and the related Results sections.

10.7717/peerj.21476/supp-5 Supplemental Information 5 Raw data matrices used for correlation and principal component analyses Raw and compiled data matrices used for Pearson correlation analysis and principal component analysis (PCA). The matrices integrate soil chemical properties, rice bran oil yield, bioactive compound measurements, elemental composition, and fatty acid variables derived from the individual supplementary datasets.
These data were used as inputs for the correlation heatmap and PCA biplots presented in Figure 6 and to support the multivariate analyses described in the Results section.

10.7717/peerj.21476/supp-6 Supplemental Information 6 Two-way ANOVA results for soil properties across salinity level and rice variety, including effect sizes The full two-way ANOVA outputs for soil properties measured in the study, including pH, SOC, TON, ECe, SAR, soil chloride, soil Fe, soil Zn, and exchangeable cations. The effects of salinity level, rice variety, and their interaction were evaluated using Type III sums of squares. Levene’s test results are also included to assess homogeneity of variance. Effect sizes are reported as partial eta squared (partial *η*^2^).

10.7717/peerj.21476/supp-7 Supplemental Information 7 Two-way ANOVA results for RBO yield, bioactive compounds, and antioxidant activity across salinity level and rice variety, including effect sizes The full two-way ANOVA outputs for rice bran oil (RBO) yield, γ-oryzanol, total carotenoids, total phenolics, total flavonoids, and antioxidant activity (DPPH). The effects of salinity level, rice variety, and their interaction were evaluated using Type III sums of squares. Levene’s test results are also included to assess homogeneity of variance. Effect sizes are reported as partial eta squared (partial *η*^2^).

10.7717/peerj.21476/supp-8 Supplemental Information 8 Two-way ANOVA results for fatty acid composition variables across salinity level and rice variety, including effect sizes The full two-way ANOVA outputs for individual and grouped fatty acid composition variables of rice bran oil, including saturated, monounsaturated, and polyunsaturated fatty acid classes and their ratios. The effects of salinity level, rice variety, and their interaction were evaluated using Type III sums of squares. Levene’s test results are also included to assess homogeneity of variance. Effect sizes are reported as partial eta squared (partial *η*^2^).

10.7717/peerj.21476/supp-9 Supplemental Information 9 Two-way ANOVA results for RBO elemental composition across salinity level and rice variety, including effect sizes The full two-way ANOVA outputs for elemental composition variables of rice bran oil (RBO), including potassium, sodium, calcium, iron, magnesium, and zinc. The effects of salinity level, rice variety, and their interaction were evaluated using Type III sums of squares. Levene’s test results are also included to assess homogeneity of variance. Effect sizes are reported as partial eta squared (partial *η*^2^).

## Additional Information and Declarations

### Field Study Permissions

The following information was supplied relating to field study approvals (*i.e.*, approving body and any reference numbers): Field sampling was conducted at rice cultivation sites along the Siao Yai River basin in Maha Sarakham and Roi Et provinces, northeastern Thailand.

### Data Availability

The following information was supplied regarding data availability: The raw data is available in the Supplemental Files. The data is available at Figshare: Manasathien, Jinnawat; Laojinda, Woraporn; Attarataya, Jakrada; Khanema, Piyanut (2026). Raw datasets on soil salinity, fatty acid composition, and antioxidant properties of rice bran oil from northeastern Thailand. figshare. Dataset. https://doi.org/10.6084/m9.figshare.31082683.v4.
